# Utilize proteomic analysis to identify potential therapeutic targets for combating sepsis and sepsis-related death

**DOI:** 10.3389/fendo.2024.1448314

**Published:** 2024-09-16

**Authors:** Tianlong Zhang, Yin Shi, Jiayue Li, Peiyao Huang, Kun Chen, Jiali Yao

**Affiliations:** ^1^ Department of Critical Care Medicine, Jinhua Hospital Affiliated to Zhejiang University, Jinhua, Zhejiang, China; ^2^ Department of Critical Care Medicine, the Fourth Affiliated Hospital of School of Medicine, and International School of Medicine, International Institutes of Medicine, Zhejiang University, Yiwu, China; ^3^ Department of Internal Medicine, Yiwu Maternity And Children Hospital, Yiwu, Zhejiang, China; ^4^ Department of Anesthesiology, the Fourth Affiliated Hospital of School of Medicine, and International School of Medicine, International Institutes of Medicine, Zhejiang University, Yiwu, China; ^5^ Department of Gastroenterology, the Fourth Affiliated Hospital of School of Medicine, and International School of Medicine, International Institutes of Medicine, Zhejiang University, Yiwu, China

**Keywords:** sepsis, plasma protein, Mendelian randomization, genetic correlation, colocalization

## Abstract

**Background:**

Sepsis is an inflammatory disease that leads to severe mortality, highlighting the urgent need to identify new therapeutic strategies for sepsis. Proteomic research serves as a primary source for drug target identification. We employed proteome-wide Mendelian randomization (MR), genetic correlation analysis, and colocalization analysis to identify potential targets for sepsis and sepsis-related death.

**Methods:**

Genetic data for plasma proteomics were obtained from 35,559 Icelandic individuals and an initial MR analysis was conducted using 13,531 sepsis cases from the FinnGen R10 cohort to identify associations between plasma proteins and sepsis. Subsequently, significant proteins underwent genetic correlation analysis, followed by replication in 54,306 participants from the UK Biobank Pharma Proteomics Project and validation in 11,643 sepsis cases from the UK Biobank. The identified proteins were then subjected to colocalization analysis, enrichment analysis, and protein-protein interaction network analysis. Additionally, we also investigated a MR analysis using plasma proteins on 1,896 sepsis cases with 28-day mortality from the UK Biobank.

**Results:**

After FDR correction, MR analysis results showed a significant causal relationship between 113 plasma proteins and sepsis. Genetic correlation analysis revealed that only 8 proteins had genetic correlations with sepsis. In the UKB-PPP replication analysis, only 4 proteins were found to be closely associated with sepsis, while validation in the UK Biobank sepsis cases found overlaps for 21 proteins. In total, 30 proteins were identified in the aforementioned analyses, and colocalization analysis revealed that only 2 of these proteins were closely associated with sepsis. Additionally, in the 28-day mortality MR analysis of sepsis, we also found that only 2 proteins were significant.

**Conclusions:**

The identified plasma proteins and their associated metabolic pathways have enhanced our understanding of the complex relationship between proteins and sepsis. This provides new avenues for the development of drug targets and paves the way for further research in this field.

## 1 Background

Sepsis, a critical condition marked by a systemic inflammatory response to infection and characterized by a high mortality rate, continues to be a formidable challenge in current clinical management and treatment (1). According to recent analyses of the global burden of disease, sepsis accounts for 19.7% of all deaths worldwide, and although the mortality rate has decreased in recent years, it remains a major threat to human health (2). Despite advances in various anti-infection and supportive care techniques (3), the high mortality rate associated with sepsis underscores the urgent need for more effective therapeutic interventions. Consequently, identifying the pathogenesis of sepsis has become crucial for developing innovative treatment strategies.

Proteomics involves the large-scale study of proteins, including their structure, function, and expression, enabling a comprehensive analysis of protein changes during sepsis (4).Proteomic analysis, a newly emerged method in recent years, has become a pivotal tool in combating sepsis. Researchers can identify and quantify thousands of proteins from patient samples, revealing the dynamic changes associated with the progression of sepsis and responses to treatment, thereby providing deep insights into the complex biological processes underlying the disease (5).Traditional proteomic studies are limited by high costs and ethical considerations in participant recruitment. Therefore, in recent years, MR analysis has been widely used for drug target development and the repurposing of existing drugs (6).

MR is a novel statistical method used to evaluate the causal effect of a factor on a disease while minimizing bias caused by confounding factors or reverse causation (7). MR analysis achieves causal inference and identifies actionable proteins by using instrumental variable data from large-scale Genome-Wide Association Studies (GWAS) and known single nucleotide polymorphisms (SNPs) associated with circulating proteins. Previous studies have used MR analysis to identify therapeutic targets for proteins related to multiple sclerosis and inflammatory bowel disease (8, 9). However, so far, no MR studies have combined GWAS and protein quantitative trait loci (pQTL) data to investigate sepsis and 28-day sepsis mortality. Therefore, the purpose of this study is to implement MR analysis to (1): identify plasma proteins as potential therapeutic targets for sepsis and 28-day sepsis mortality, and (2) determine potential metabolic pathways and associated protein functions that may contribute to understanding the mechanisms underlying sepsis and 28-day sepsis mortality. Our findings could lay the foundation for future research directions in sepsis treatment and efforts to reduce sepsis mortality.

## 2 Materials and methods

### 2.1 Study design

**Figure 1:**
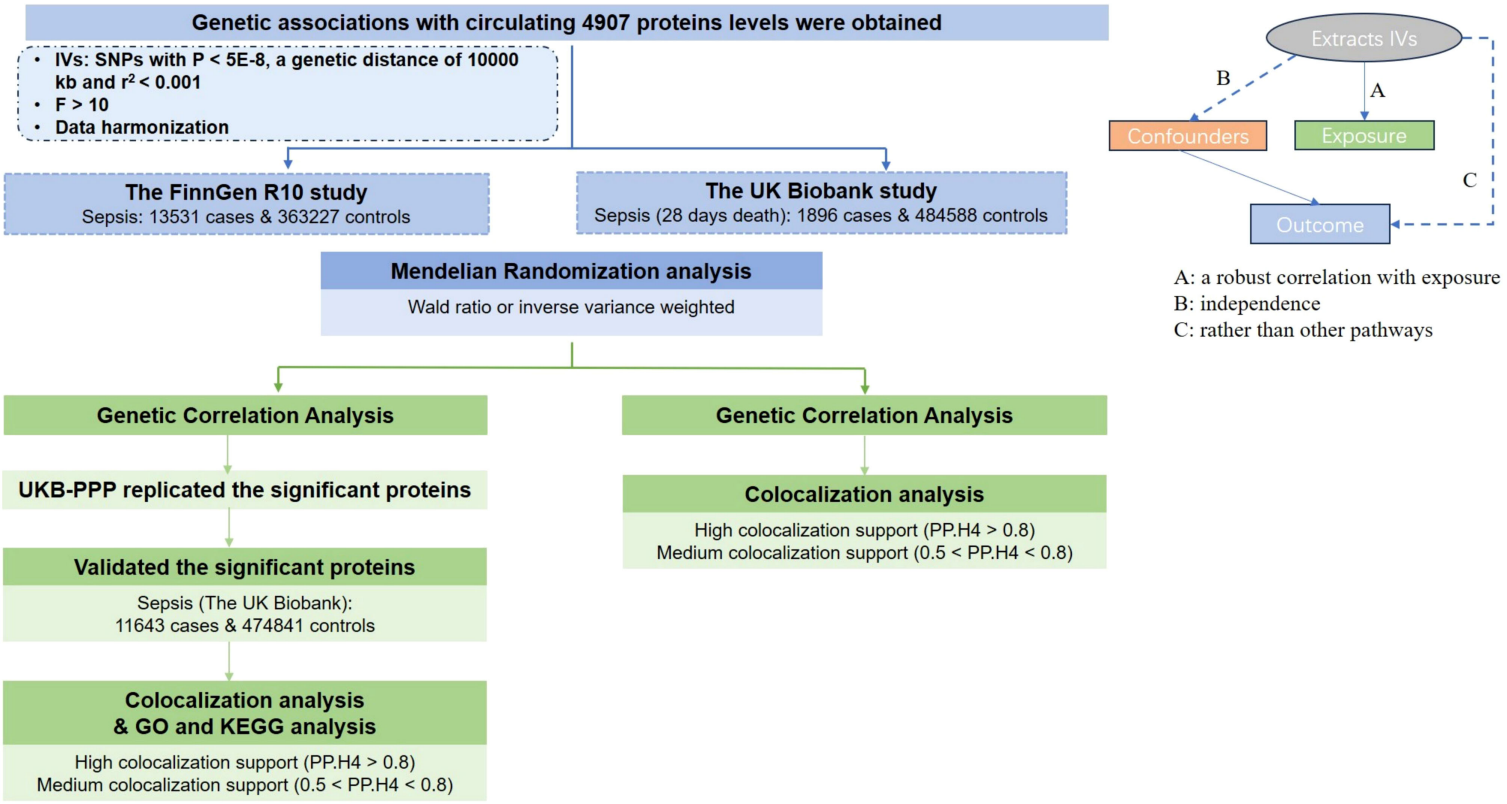
A schematic overview of the study design.

The MR study was designed to investigate the causal relationship between plasma proteins and sepsis. **Figure 1** provides a schematic overview of the study design. This study follows the reporting guidelines as specified in STROBE-MR (**Additional File**).The MR approach must satisfy three key conditions (**Figure 1**): (A) The genetic variants selected as instrumental variables (IVs) must be strongly correlated with plasma proteins; (B) The genetic instruments must be unrelated to sepsis outcomes and independent of potential confounding factors; (C) The genetic variants should influence sepsis risk specifically through plasma proteins rather than through other pathways.

### 2.2 GWAS data sources

In the preliminary analysis, summary-level data for 4,907 plasma proteins were obtained from the GWAS of 35,559 Icelanders (10). For the proteins identified as being associated with sepsis in the initial analysis, we used another independent protein GWAS source (N = 54,219), the UK Biobank Pharma Proteomics Project (UKB-PPP) (11), for replication. Sepsis outcome data were obtained from 13,531 cases and 363,227 controls of European ancestry in the FinnGen R10 Biobank (12). Significant proteins identified from the preliminary analysis were validated using data from 11,643 sepsis cases and 474,841 controls of European ancestry in the UK Biobank (13).The studies conducted by these consortia received approval from local research ethics committees and institutional review boards, with all participants giving written informed consent.

### 2.3 Instrumental variables selection

We selected SNPs associated with plasma proteins at genome-wide significance levels (P < 5 × 10^-8^) (14) as IVs for the proteins. All IVs were clumped for linkage disequilibrium (LD) (R^2^< 0.001; distance = 10,000 kb) to reduce the impact of correlations between SNPs. Additionally, R^2^ and F-statistics were calculated to assess the strength of the IVs, and IVs with an F-statistic greater than 10 were selected.

### 2.4 MR analysis

Herein, a two-sample MR(TSMR) analysis was utilized to evaluate the causal relationship between plasma proteins and sepsis, as well as 28-day mortality in sepsis. Subsequently, the fixed-effect inverse variance-weighted (IVW) method and the Wald ratio method were employed as the primary MR analyses. For proteins detected by multiple SNPs, we used the IVW method, whereas for proteins detected by a single SNP, we used the Wald ratio method. The Cochran Q test was used to assess heterogeneity in causal effects, and the MR-Egger intercept was employed to evaluate horizontal pleiotropy. P-values below 0.05 in these tests typically indicate the presence of heterogeneity or pleiotropy (15). To identify more actionable plasma proteins during multiple comparisons, we applied false discovery rate (FDR) correction, setting the FDR threshold at <0.2. During external validation in sepsis, we used a nominal P-value threshold of less than 0.05 to determine the statistical significance of these significant proteins.

The MR analysis analyses were performed using R software version 4.3.3 and the TwoSampleMR package.

### 2.5 Genetic correlation analysis

Linkage Disequilibrium Score Regression (LDSC) was used to assess shared polygenic structures between traits, with LD scores calculated from European ancestry samples of the 1000 Genomes Project serving as the reference group. This method evaluates genetic correlations from GWAS summary statistics without introducing bias from sample overlap (16). In an effort to identify plasma proteins more closely associated with sepsis, we conducted genetic correlation analysis on the significant proteins identified in the MR analysis. By not applying corrections and relaxing the P-value threshold to 0.1, we aimed to capture more proteins with genetic correlations.

### 2.6 Colocalization analysis

Colocalization analysis was further conducted in this study to examine plasma proteins. We used the coloc R package to strengthen the genetic study results by finding evidence of shared genetic variants associated with both plasma proteins and sepsis. The Bayesian analysis evaluates support for five mutually exclusive hypotheses (17): H0, where a genetic variation is not associated with any trait; H1, associated only with the first trait; H2, associated only with the second trait; H3, associated with both traits but with different causal variants; and H4, associated with both traits and sharing the same causal variant (19). We calculated the posterior probabilities (PP) for each hypothesis and considered strong evidence for colocalization when PP.H4 was greater than 0.8. Medium evidence for colocalization was defined as 0.5 < PP.H4 < 0.8.

### 2.7 Functional enrichment analysis and pathway analysis

We utilized the KEGG Orthology-Based Annotation System (KOBAS) to perform Gene Ontology (GO) and Kyoto Encyclopedia of Genes and Genomes (KEGG) enrichment analyses (18). These analyses aimed to provide a better understanding of the biological functions and metabolic pathways of similarly expressed proteins. GO analysis studies the commonalities of genes in terms of biological processes (BP), cellular components (CC), and molecular functions (MF). It assesses the enrichment of genes within each GO annotation by comparing the analyzed genes to the reference genome, thus producing the results of the enrichment analysis. KEGG enrichment analysis primarily focuses on the enrichment of genes within metabolic pathways. The enrichment analysis in this study was conducted on proteins with a multiple adjusted P-value < 0.2.

### 2.8 Protein-protein interaction network analysis

We used GeneMANIA (http://genemania.org) (19) to analyze the protein-protein interaction (PPI) network of the significant proteins we identified.

### 2.9 Additional analysis

Additionally, we investigated 28-day mortality in sepsis, using data from 1,896 cases and 484,588 controls of European ancestry from the UK Biobank (13).Due to potential overlap in the population, we did not perform replication in the UKB-PPP, and currently, there are no other GWAS studies available on 28-day mortality in sepsis, so we did not validate the 28-day mortality outcomes for sepsis.

## 3 Results

### 3.1 Plasma proteins and sepsis

**Figure 2:**
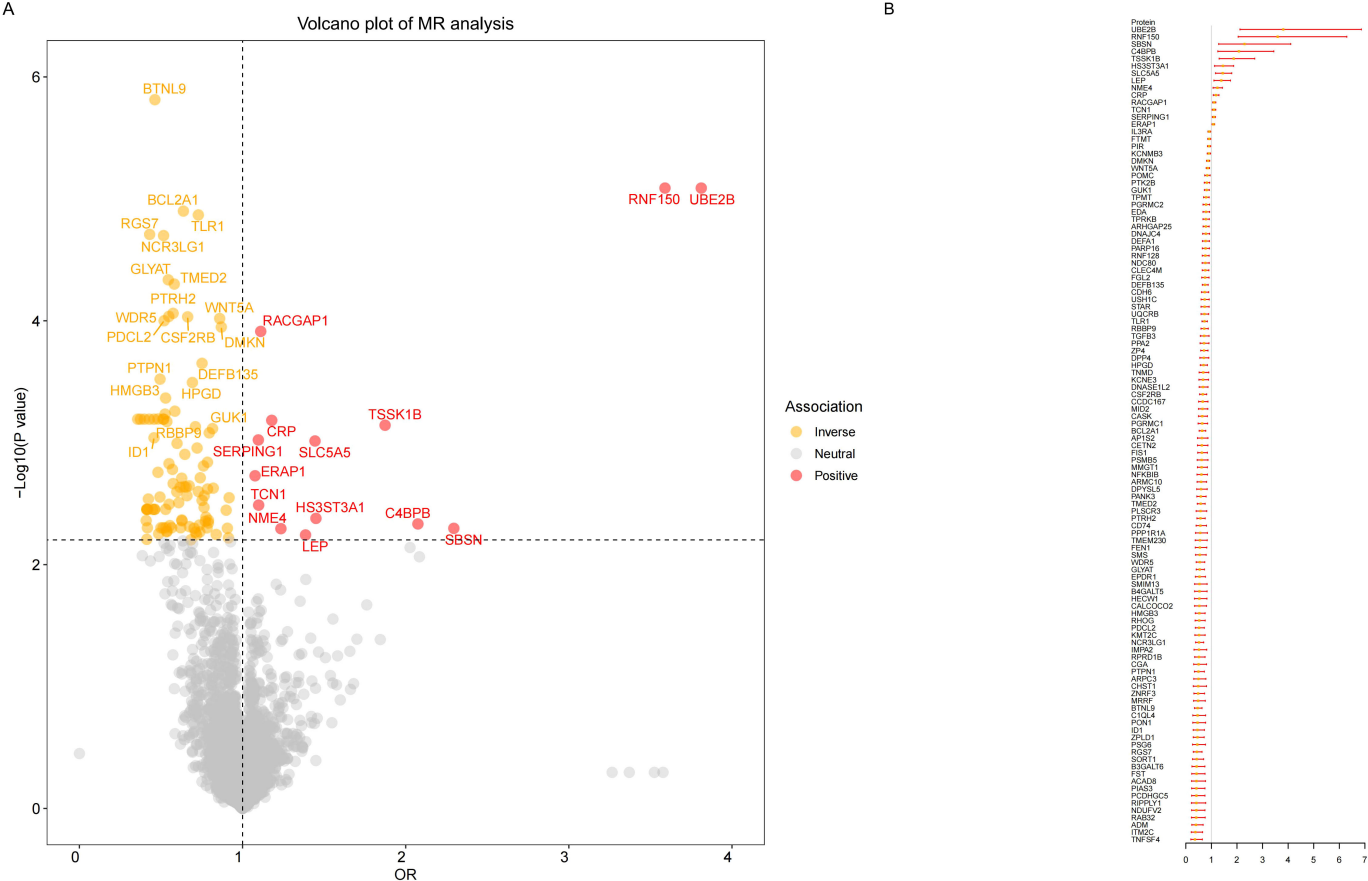
Result of MR analysis on the associations between plasma proteins and the risk of sepsis. **(A)** The volcano plot shows the results of the proteome-wide MR analysis of sepsis using the initial protein data. **(B)**The forest plot shows the MR associations between 113 significant plasma proteins and sepsis risk identified using the initial protein data.

**Figure 3:**
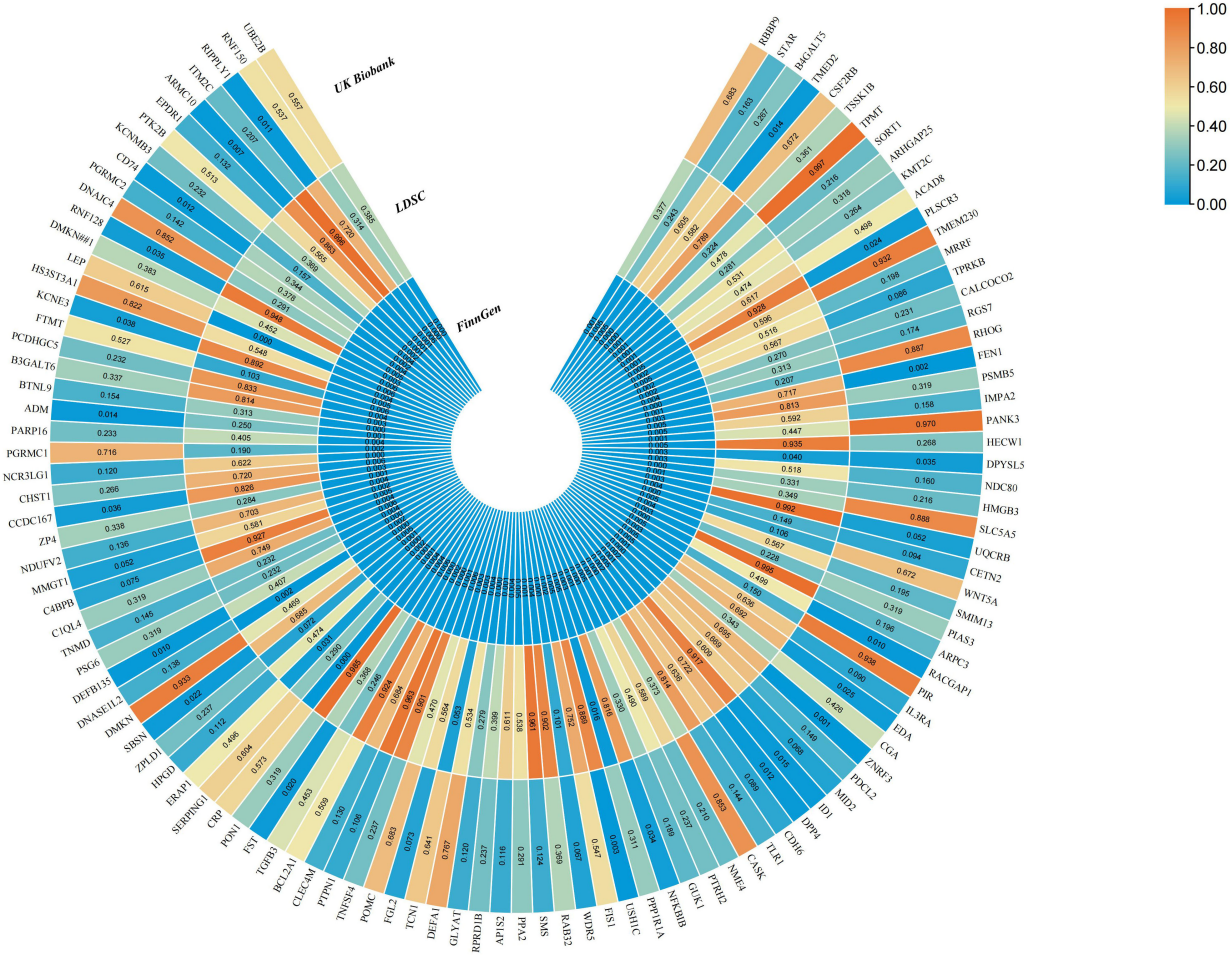
The MR results of sepsis for 113 plasma proteins in the FinnGen cohort and UK Biobank, as well as the genetic correlation analysis results of these plasma proteins.

**Figure 4:**
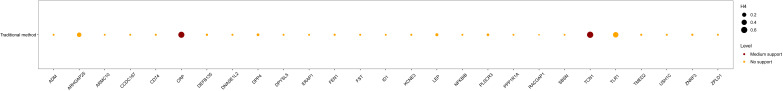
The colocalization analysis of the identified 30 proteins. Medium evidence for colocalization was defined as 0.5 < H4 < 0.8, while strong evidence was defined as H4 > 0.8.

Our MR results indicated suggestive associations between 252 plasma proteins (P < 0.05) and sepsis (**Supplementary Tables S1, S2**). After FDR correction, we observed statistically significant associations between 113 plasma proteins and sepsis. Among these proteins, 99 were protective, with butyrophilin-like protein 9 (BTNL9) (OR: 0.463, 95% CI: 0.338–0.634, FDR= 5.50×10^-3^) being the most notable. Additionally, there were 14 risk proteins that exacerbate sepsis, with RING finger protein 150 (RNF150) (OR: 3.589, 95% CI: 2.047–6.293, FDR= 9.68×10^-3^) being the most significant. **Figure 2** presents the 113 significant associations, with details provided in **Supplementary Table S3**.Genetic correlation analysis of these 113 proteins revealed that only leptin (LEP)(rg = 0.562, P = 7.28×10^-6^), c-reactiveprotein (CRP) (rg = 0.548, P = 1.32×10^-4^),deoxyribonuclease-1-like 2 (DNASE1L2)(rg = 0.478, P = 2.40×10^-3^), protein phosphatase 1 regulatory subunit 1A (PPP1R1A)(rg = 0.396, P =0.016),endoplasmic reticulum aminopeptidase 1 (ERAP1) (rg = 0.246, P =0.031), dihydropyrimidinase-relatedprotein 5 (DPYSL5)(rg = 0.417, P =0.040), transcobalamin-1 (TCN1) (rg = 0.260, P =0.053), and zona pellucida-like domain-containing protein 1 (ZPLD1) (rg = 0.420, P =0.072) had genetic correlations with sepsis(**Supplementary Table S4**). Subsequently, we replicated these 113 plasma proteins in the UKB-PPP and found that only toll-like receptor 1 (TLR1) (P =0.021), rho GTPase-activating protein 25 (ARHGAP25) (P =0.013), TCN1 (P =7.16×10^-3^), and LEP (P =5.74×10^-3^), were closely associated with sepsis (**Supplementary Table S5**). We also validated these 113 proteins in the sepsis outcomes of the UK Biobank and found overlaps withE3 ubiquitin-protein ligase ZNRF3 (ZNRF3) (P =1.03×10^-3^),flap endonuclease 1 (FEN1)(P =1.81×10^-3^), harmonin (USH1C) (P =2.98×10^-3^), armadillo repeat-containing protein 10 (ARMC10) (P =6.89×10^-3^), racGTPase-activating protein 1 (RACGAP1)(P =9.65×10^-3^), beta-defensin 135 (DEFB135) (P =0.010), protein ripply1(RIPPLY1)(P =0.011),dipeptidyl peptidase 4 (DPP4) (P =0.012), HLA class II histocompatibility antigen gamma chain (CD74) (P =0.012), transmembrane emp24 domain-containing protein 2 (TMED2)(P =0.014), pro-adrenomedullin (ADM) (P =0.014), DNA-binding protein inhibitor ID-1 (ID1) (P =0.015),follistatin (FST)(P =0.020), suprabasin (SBSN)(P =0.022), phospholipid scramblase 3 (PLSCR3) (P =0.024), ectodysplasin-A (EDA) (P =0.025), NF-kappa-B inhibitor beta (NFKBIB)(P =0.034), E3 ubiquitin-protein ligase RNF128 (RNF128)(P =0.035), DPYSL5(P =0.035),coiled-coil domain-containing protein 167 (CCDC167) (P =0.036),and potassium voltage-gated channel subfamily E member 3 (KCNE3) (P =0.038)(**Supplementary Table S6**). **Figure 3** illustrates the aforementioned associations. We then performed colocalization analysis on a total of 30 proteins identified through genetic correlation analysis and overlapping proteins, finding that only CRP and TCN1 had moderate support for colocalization (**Supplementary Table S7**, **Figure 4**, **Supplementary Figures S1, S2**). MR-Egger regression did not reveal evidence of horizontal pleiotropy (P > 0.05). Cochran’s Q test indicated heterogeneity for CRP (P = 0.013), while no significant heterogeneity was found for the other proteins (**Supplementary Table S3**). Due to the limited number of SNPs for some proteins, tests for pleiotropy and heterogeneity could not be conducted, but this does not affect the main objectives of our study.

### 3.2 Plasma proteins and sepsis (28-day mortality)

MR results showed that 111 plasma proteins (P < 0.05) were associated with 28-day mortality in sepsis (**Supplementary Table S8**). After FDR correction, only Apolipoprotein A-I (APOA1) (OR: 0.710, 95% CI: 0.598–0.844, FDR=0.169) and homeodomain-interacting protein kinase 3 (HIPK3) (OR: 0.149, 95% CI: 0.059–0.377, FDR=0.172) remained statistically significant, and both were identified as protective proteins with no detected heterogeneity or pleiotropy. Unfortunately, these two proteins did not show significant results in the genetic correlation analysis and did not receive support from colocalization (**Supplementary Table S9**).

### 3.3 GO+KEGG analysis

**Figure 5:**
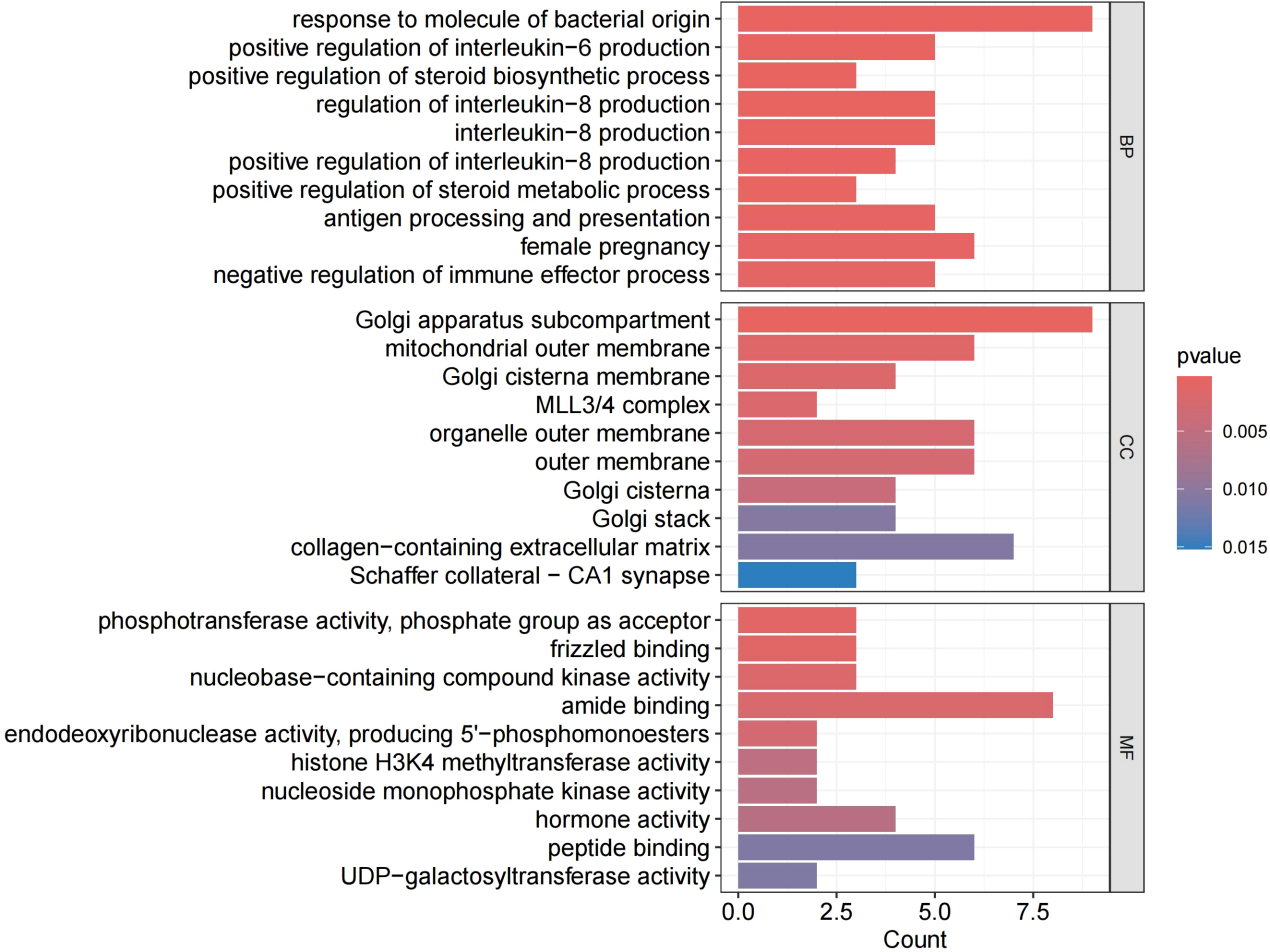
GO enrichment analysis. BP, biological processes; CC, cellular components; MF, molecular functions.

**Figure 6:**
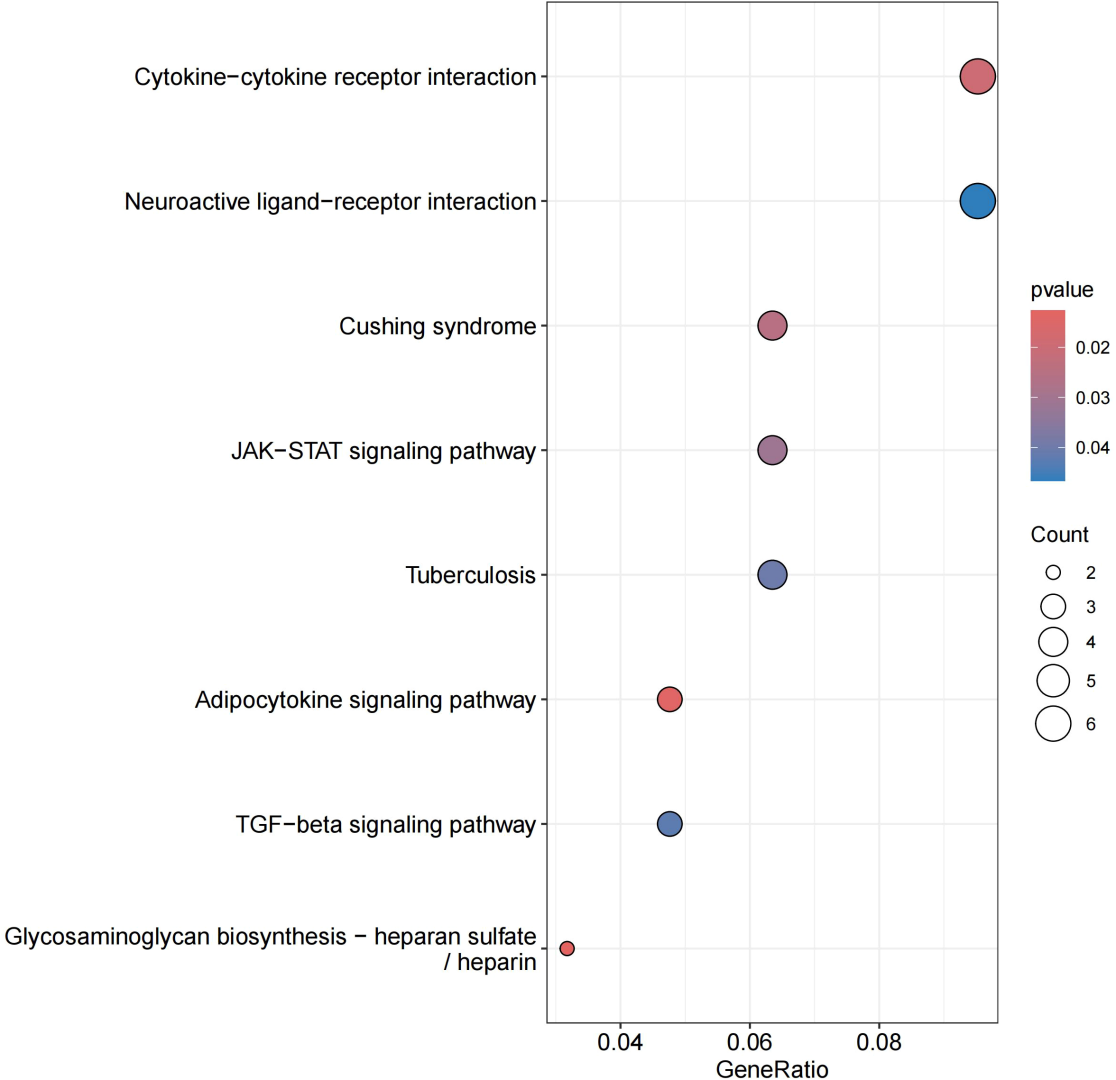
KEGG enrichment analysis.

We performed GO and KEGG analysis on the 113 significant plasma proteins identified in sepsis. GO annotation results showed that the BP category was mainly enriched for response to molecule of bacterial origin, positive regulation of interleukin-6 production, regulation of interleukin-8 production, etc. The CC category included golgi apparatus sub compartment, mitochondrial outer membrane, organelle outer membrane, etc. The MF category primarily involved phosphotransferase activity, phosphate group as acceptor, frizzled binding, amide binding, etc (**Supplementary Table S10**, **Figure 5**, **Supplementary Figure S3**). KEGG enrichment analysis indicated that these proteins are primarily involved in the cytokine-cytokine receptor interaction, adipocytokine signaling pathway, and cushing syndrome, etc (**Supplementary Table S11**, **Figure 6**).Given that only two proteins were identified for 28-day mortality in sepsis, GO and KEGG analyses were not performed.

### 3.4 PPI network analysis

The PPI network analysis in **Supplementary Figure S4** reveals 30 genes that interact with each other.

## 4 Discussion

Due to the rapid advancements in proteomics research in recent years, there is a better understanding of developing new drugs for sepsis. Most proteomic studies are observational, which can demonstrate associations with sepsis but cannot establish causality. We used MR to investigate the causal impact of circulating plasma proteins on sepsis and 28-day mortality, providing critical preclinical insights for drug development. In our MR analysis, post-FDR correction, we identified 113 plasma proteins strongly linked to sepsis and 2 closely associated with 28-day sepsis mortality. We then performed a genetic correlation analysis to identify which of the 113 proteins were most strongly associated with sepsis. The results indicated that LEP, CRP, DNASE1L2, PPP1R1A, ERAP1, DPYSL5, TCN1, and ZPLD1 had genetic correlations with sepsis. We replicated the 113 plasma proteins in the UKB-PPP and identified TLR1, ARHGAP25, TCN1, and LEP were strongly associated with sepsis. We also validated the sepsis outcomes of these 113 proteins in the UKB, identifying overlap with 21 proteins. In the subsequent colocalization analysis, only CRP and TCN1 showed medium support for colocalization. Additionally, GO and KEGG enrichment analyses, along with PPI network analysis, were performed on the 30 identified plasma proteins to elucidate their biological significance of these drug targets. These insights deepen our understanding of sepsis’s molecular mechanisms and facilitate the identification of biomarkers for early diagnosis and monitoring, thereby paving the way for the development of precision therapies and advanced early diagnostic techniques.

Among the 30 sepsis-related proteins, TCN1, LEP, CRP, ERAP1, RACGAP1, and SBSN were associated with an elevated risk sepsis, while TLR1, NFKBIB, ARHGAP25, PPP1R1A, DPYSL5, DNASE1L2, ZPLD1, TMED2, DEFB135, ZNRF3, ADM, ID1, ARMC10, CD74, PLSCR3, CCDC167, RNF128, FST, KCNE3, DPP4, USH1C, RIPPLY1, FEN1, and EDA were correlated with a reduced risk of sepsis.

In this study, we identified Transcobalamin-1 (TC-I) as a significant sepsis target, and its levels were positively associated with the risk of sepsis. TC-I, a 60-70 kDa R-binder protein encoded by the TCN1 gene, serves as a cobalamin carrier, which regulates cobalamin homeostasis (20–22). Our results validated TC-I as a risk protein for sepsis through both genetic correlation analysis and colocalization methods. Previous studies have linked the TCN1 gene to sepsis mortality (23), elevated TCS (TC-I and TC-II) and increased unsaturated B12 binding capacity are observed in cobalamin deficiency during sepsis (24), and the elevation of TCS in the immune-inflammatory response is a consequence of NF-kappaB activation (24). This suggests that TC-I’s association with increased sepsis risk may be related to cobalamin deficiency. Additionally, TC-I is elevated in other inflammatory conditions, such as asthma, inflammatory bowel disease, and cancer-associated inflammation. In asthma patients, TCN1 levels were elevated in induced sputum supernatant and correlated with the fraction of exhaled nitric oxide, IgE, PC-20, forced expiratory volume in the first second (FEV1)% predicted, FEV1/FVC, and several cytokines (IL-4, IL-5, IL-10, IL-13, MUC5AC) (25). In inflammatory bowel disease, TCN1 expression was elevated in refractory ulcerative colitis and correlated with the severity of intestinal inflammation in Crohn’s disease (26). In gastric cancer, strong immune reactivity of the TCN1 protein was significantly associated with tumor invasion depth, regional lymph node involvement, and tumors larger than 5 cm in diameter. Elevated TCN1 expression also indicated a poor clinical prognosis (27). Thus, TC-I may serve as a key risk biomarker and therapeutic target, but further validation is required.

Leptin, produced by adipocytes and encoded by the LEP gene, regulates food intake, energy expenditure, and is vital for body mass control and metabolism (28), it also has notable proinflammatory effects (29). We performed KEGG enrichment analysis to elucidate the biological functions and metabolic pathways of similarly expressed proteins. The two KEGG pathways in our prediction list—cytokine-cytokine receptor interaction and neuroactive ligand-receptor interaction—both describe the mechanism associated biological processes and pathways have been reported to participate in sepsis (30–32), and leptin acts through a transmembrane receptor in these pathways (28, 33, 34). Our findings suggest leptin is a likely causal protein, with elevated levels positively correlating with sepsis severity scores (29).Thus, investigating leptin’s role in sepsis is crucial.

CRP, identified through genetic correlation analysis and medium colocalization support, is linked to higher sepsis risk and is widely used in sepsis (35). Endoplasmic reticulum aminopeptidase 1 (ARTS1), encoded by ERAP1, processes peptides for major histocompatibility complex class I presentation in the endoplasmic reticulum, and secreted ERAP1 boosts the expression of pro-inflammatory cytokines (36). Its role as a sepsis risk protein remains unverified and needs further study.

The GO enrichment analysis showed that these 113 genes are significantly linked to the response to molecules of bacterial origin in biological processes, which are associated with sepsis, with NF-κB signaling confirmed to include this feature (37, 38). We hypothesize that NF-κB inhibitor beta (IκB-β) also plays a role in this process. Our results support this hypothesis. IκB-β, encoded by the NFKBIB gene, is a key member of the mammalian IκB proteins family that primarily inhibits NF-κB activity through protein-protein interactions between IκB proteins and NF-κB dimers in the cytosol (39). The NF-κB pathway recruits M1 macrophages to release cytokines (TNF-α, IL-6), which exacerbate cytokine storms and sepsis-induced ALI/ARDS (40). Previous research shows aspirin effectively treats sepsis by inhibiting NF-κB mobilization in stimulated endothelial cells (41, 42). IκB-β has a similar role in sepsis, as evidenced by Wang et al (43), who reported that overexpression of hypo-phosphorylated IκB-β at Ser313 protects the heart against sepsis. Thus, IκB-β emerges as a protective protein with potential as a therapeutic candidate.

We also provided evidence through genetic correlation analysis and results from the UKB validation set that dihydropyrimidinase-related protein 5 (DPYL5) levels are negatively correlated with the risk of sepsis. DPYL5, encoded by the DPYSL5 gene, has been linked to a negative correlation with the NF-κB signaling pathway (44). Given NF-κB’s role in promoting sepsis, DPYSL5 may potentially inhibit sepsis progression. This hypothesis aligns with our findings, but the link between DPYSL5 and sepsis is scarcely documented, suggesting its potential as a novel target for further research.

In sepsis research, the 28-day mortality rate is the most relevant endpoint for evaluating therapeutic efficacy (45). We found HIPK3 and Apolipoprotein A-I (ApoA-I) linked to reduced 28-day sepsis mortality. HIPK3, encoded by the HIPK3 gene, may slow sepsis by inhibiting the JNK/c-Jun pathway, as reported by Liu et al (46). ApoA-I, encoded by the APOA1 gene, is the principal protein in high-density lipoprotein cholesterol particles in plasma (47). Several studies indicate that ApoA-I provides protection against sepsis, with reduced ApoA-I levels commonly observed in inflammation and sepsis, inversely correlating with disease severity in cirrhotic patients with severe sepsis (47–49). Both HIPK3 and ApoA-I are protective proteins and potential targets for reducing sepsis 28-day mortality.

One strength of this investigation is that we are the first to use MR analyses to exploit genetic variants for discovering new therapeutic targets for plasma proteins in sepsis and 28-day sepsis mortality. To address potential external confounding factors that often affect observational studies and to clarify the direct relationship between exposure and outcome, we employed several methods, including TSMR, genetic correlation analysis, and colocalization analyses. Additionally, we validated our findings using data from multiple European population databases, including protein data from Iceland and UKB-PPP, as well as sepsis data from FinnGen and UK Biobank, ensuring the reproducibility of our results in future studies. Another strength is that we focused our analysis on individuals of European ancestry, thereby minimizing population stratification bias.

Several limitations should be noted. First, as our study focuses on European populations, further research is needed to confirm whether these findings apply to other populations, such as Asians. Second, by limiting our analysis to available proteins, we may have missed other therapeutic targets. Third, although we identified 30 genes through various analyses, none fully overlapped, indicating population bias and complicating drug target identification. Fourth, caution is required when interpreting PP.H4 in colocalization, as low values don’t necessarily rule out colocalization, particularly when PP.H3 is also low due to limited power. Finally, since plasma protein levels are influenced by non-genetic factors, and our genetic findings have yet to be validated in clinical or animal models, it is crucial to underscore the importance of future epidemiological studies to confirm our results.

## 5 Conclusions

In summary, this study identified numerous associations between plasma proteins and the risk of sepsis and 28-day sepsis mortality using an integrated genetic approach. By combining data from various databases, a total of 30 genes were prioritized as potential drug targets for sepsis, enhancing our understanding of the complex relationship between proteins and sepsis. This provides new avenues for drug target development and paves the way for further research in this field, although these findings still need validation in future trials.

## Data Availability

The datasets presented in this study can be found in online repositories. The names of the repository/repositories and accession number(s) can be found in the article/**Supplementary Material**.
