# Supplementary material for: Utilize proteomic analysis to identify potential therapeutic targets for combating sepsis and sepsis-related death
Source: Front Endocrinol (Lausanne). 2024 Sep 16;15:1448314. doi: 10.3389/fendo.2024.1448314 (PMC11463698; doi:10.3389/fendo.2024.1448314)

Figure S1. The colocalization results of the CRP gene (PP.H4=0.59). The r^2^ value indicates the linkage disequilibrium (LD) between the variants and the top SNPs.


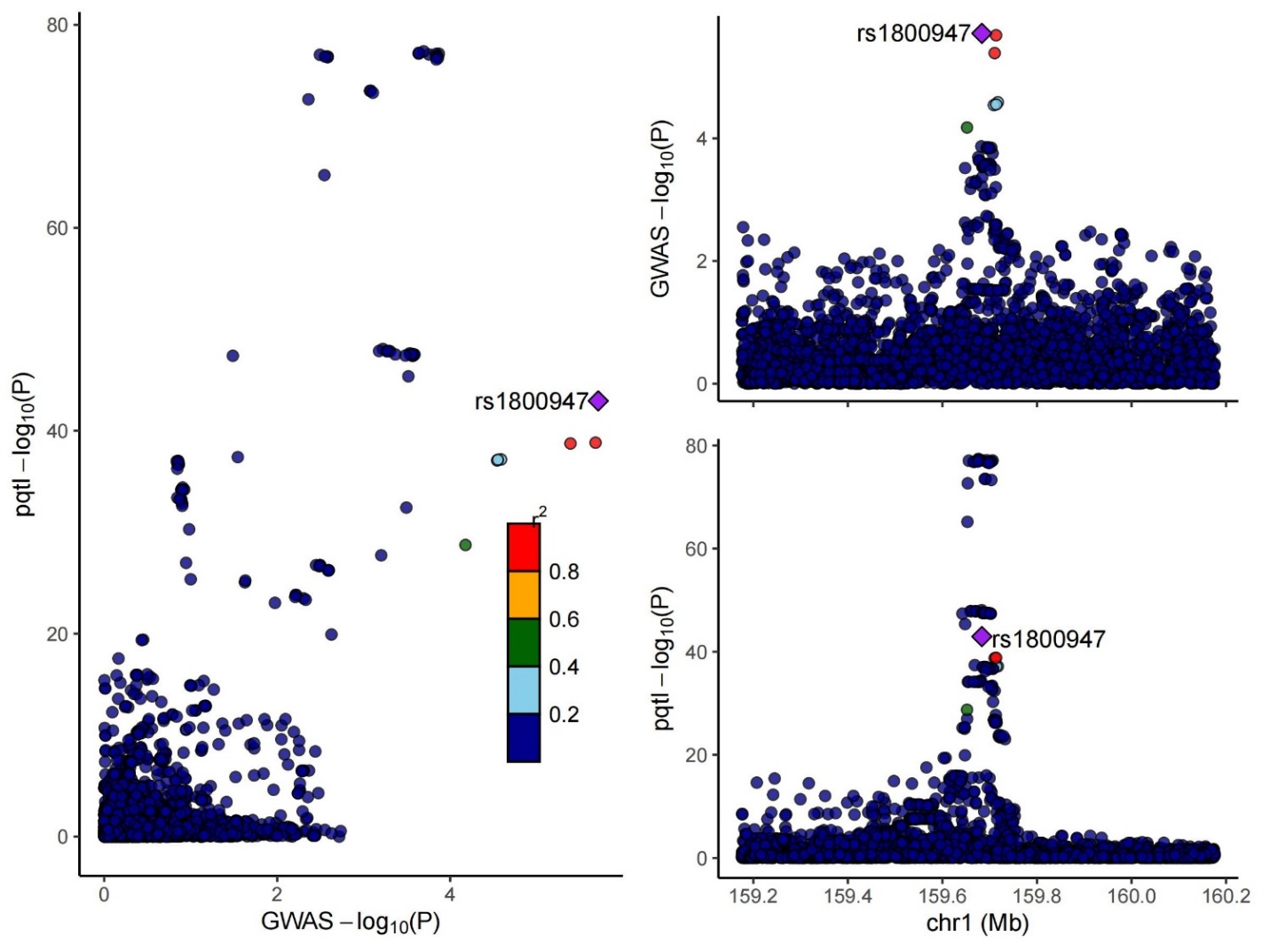


Figure S2. The colocalization results of the TCN1 gene (PP.H4=0.63). The r^2^ value indicates the LD between the variants and the top SNPs.


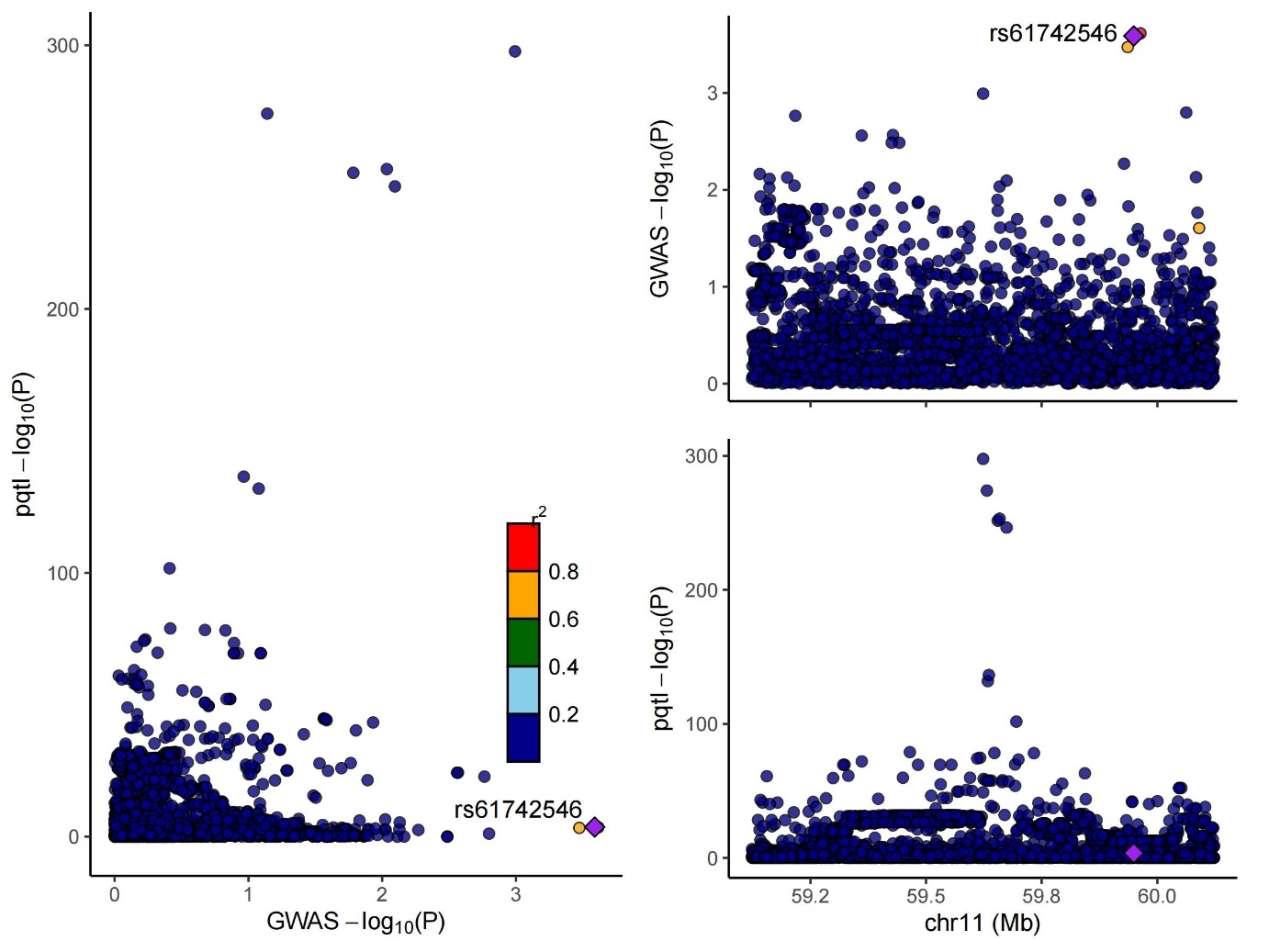


Figure S3. The circular GO enrichment analysis plot, with GO ID numbers referenced in Table S10.


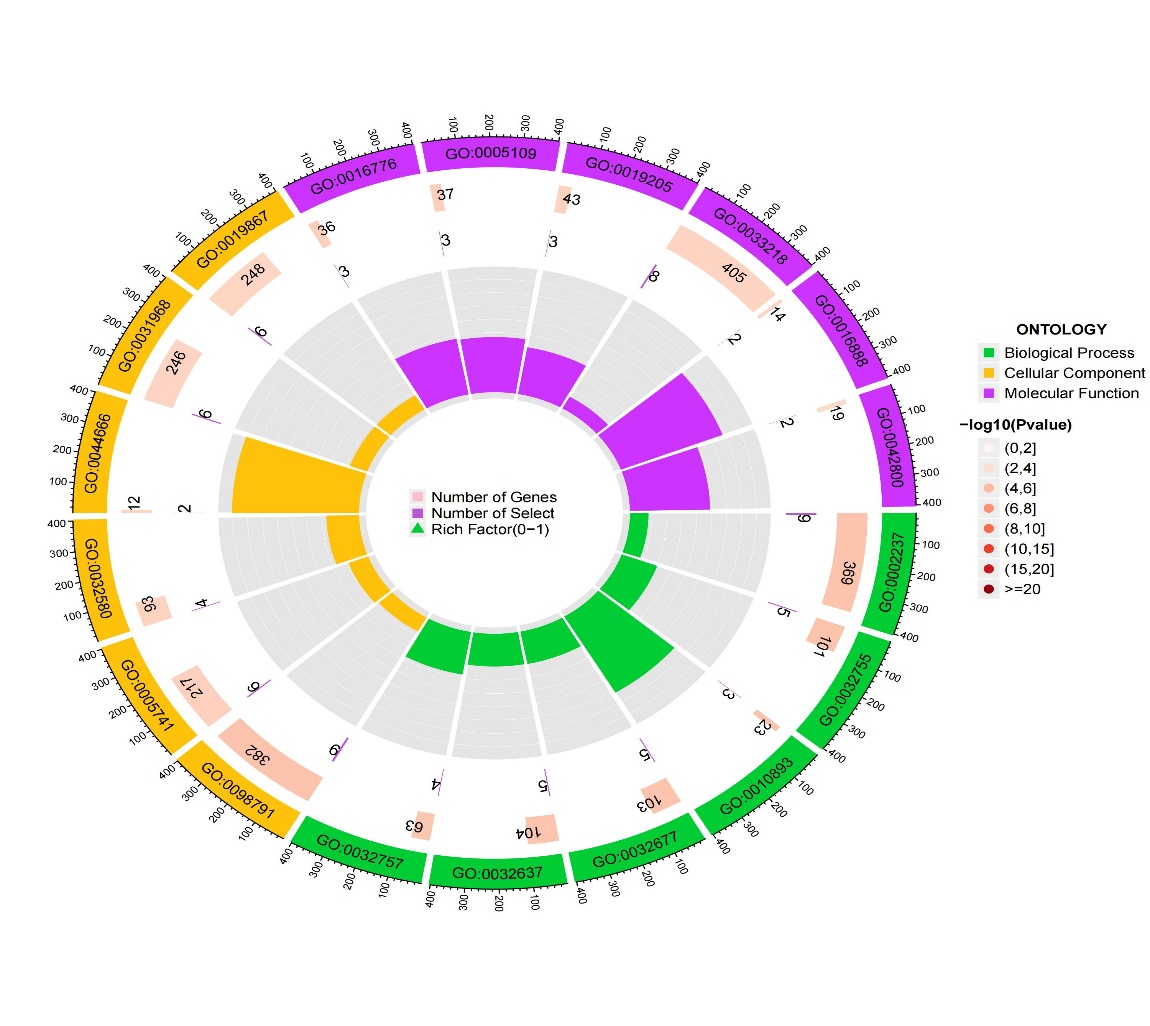


Figure S4. Protein-Protein Interaction network analysis of 30 genes


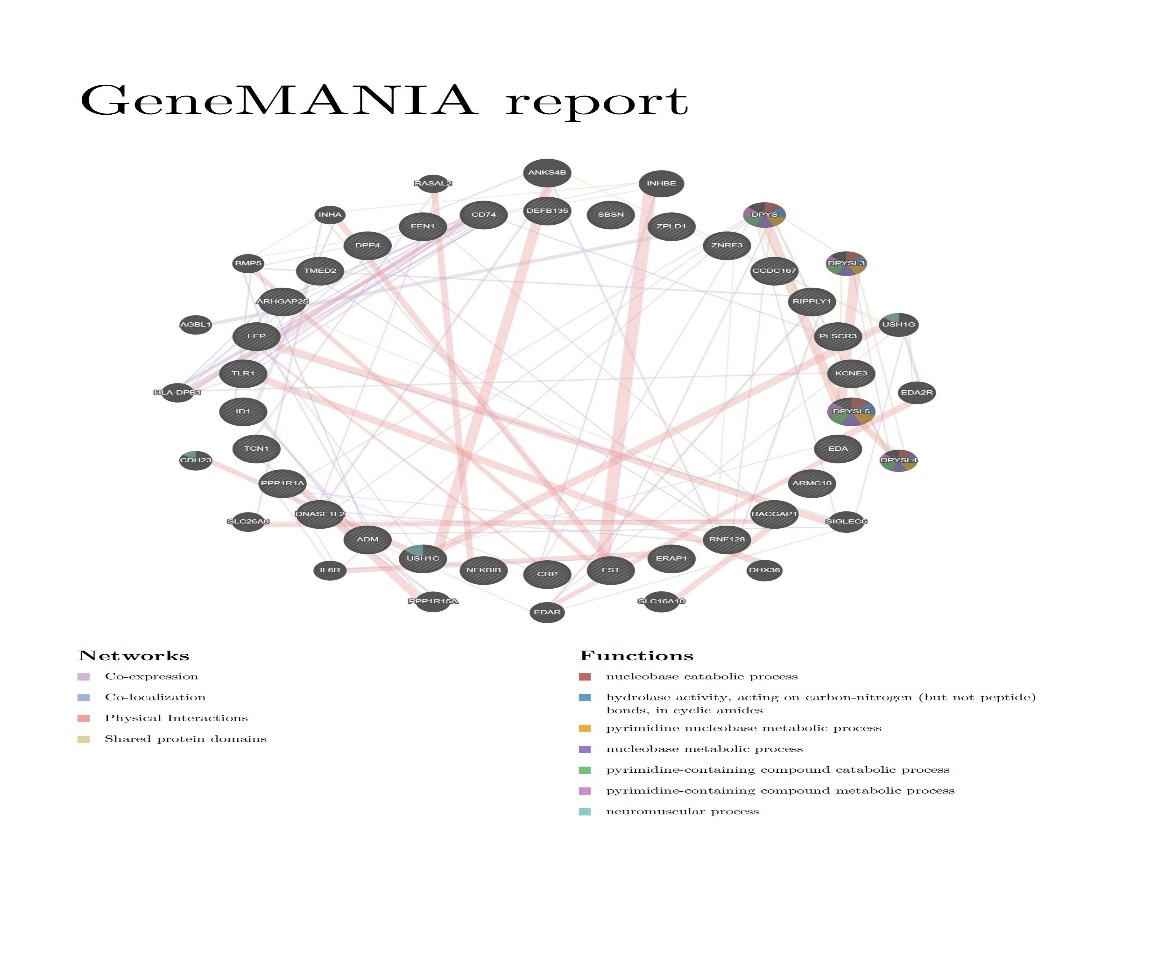

Supplement: Supplementary file 1 [file DataSheet1.docx]
