# Supplementary material for: Utilize proteomic analysis to identify potential therapeutic targets for combating sepsis and sepsis-related death
Source: Front Endocrinol (Lausanne). 2024 Sep 16;15:1448314. doi: 10.3389/fendo.2024.1448314 (PMC11463698; doi:10.3389/fendo.2024.1448314)
Supplement: Supplementary file 3 [file DataSheet3.docx]

**Additional File 1.**

STROBE-MR checklist.

| Item No. | Section | Checklist item | Page No. | Relevant text from manuscript |
| --- | --- | --- | --- | --- |
| 1 | TITLE and ABSTRACT | Indicate Mendelian randomization (MR) as the study’s design in the title and/or the abstract if that is a main purpose of the study | 2 | We employed proteome-wide Mendelian randomization (MR), genetic correlation analysis, and colocalization analysis to identify potential targets for sepsis and sepsis-related death. |
|  | INTRODUCTION |  |  |  |
| 2 | Background | Explain the scientific background and rationale for the reported study. What is the exposure? Is a potential causal relationship between exposure and outcome plausible? Justify why MR is a helpful method to address the study question | 3-5 | Sepsis, a critical condition marked by a systemic inflammatory response to infection and characterized by a high mortality rate, continues to be a formidable challenge in current clinical management and treatment. Proteomic analysis, a newly emerged method in recent years, has become a pivotal tool in combating sepsis. Therefore, the purpose of this study is to implement MR analysis to: (1) identify plasma proteins as potential therapeutic targets for sepsis and 28-day sepsis mortality, and (2) determine potential metabolic pathways and associated protein functions that may contribute to understanding the mechanisms underlying sepsis and 28-day sepsis mortality. |
| 3 | Objectives | State specific objectives clearly, including pre-specified causal hypotheses (if any). State that MR is a method that, under specific assumptions, intends to estimate causal effects | 5 | We performed a Mendelian randomization analysis to complement observational results. This study aims to evaluate the causal relationship between plasma proteins and sepsis. We assessed the risk of two different sepsis phenotypes, including the occurrence of sepsis and 28-day mortality due to sepsis. Similarly, we identified 4,907 proteins as the exposures of interest and extracted instrumental variables. |
|  | METHODS |  |  |  |
| 4 | Study design and data sources | Present key elements of the study design early in the article. Consider including a table listing sources of data for all phases of the study. For each data source contributing to the analysis, describe the following: |  |  |
|  | a) | Setting: Describe the study design and the underlying population, if possible. Describe the setting, locations, and relevant dates, including periods of recruitment, exposure, follow-up, and data collection, when available. | 5  5-6 | See Figure 1 for a schematic summary of the study design.  Exposure: In the preliminary analysis, summary-level data for 4,907 plasma proteins were obtained from the GWAS of 35,559 Icelanders. The UK Biobank Pharma Proteomics Project (UKB-PPP), for replication.  Outcome: We used publicly available summary statistics from two independent cohorts of European ancestry: UK Biobank and FinnGen (R10 biobank) (Table 1). Cases and controls were defined based on ICD10-codes ( Figure 1). |
|  | b) | Participants: Give the eligibility criteria, and the sources and methods of selection of participants. Report the sample size, and whether any power or sample size calculations were carried out prior to the main analysis | 5-6 | Exposure: Supplement Table 1.  Outcome: Sepsis and 28-day mortality in sepsis |
|  | c) | Describe measurement, quality control and selection of genetic variants | 6 | The lowest F-statistic value recorded during the validity tests was 29.71. (Supplement Table1 ). Only independent SNPs (r² = 0.001; distance = 10,000 kb) that are strongly associated (P < 5 × 10⁻⁸) with the blood levels of each protein were used in the main analyses. (Supplement Table1). |
|  | d) | For each exposure, outcome, and other relevant variables, describe methods of assessment and diagnostic criteria for diseases | 6  5-6 | Exposure: Supplement Table1.  Outcome: Cases and controls were defined based on ICD10-codes. |
|  | e) | Provide details of ethics committee approval and participant informed consent, if relevant | 6 | The studies conducted by these consortia received approval from local research ethics committees and institutional review boards, with all participants giving written informed consent. |
| 5 | Assumptions | Explicitly state the three core IV assumptions for the main analysis (relevance, independence and exclusion restriction) as well assumptions for any additional or sensitivity analysis | 5  7 | Three key assumptions explained  Sensitivity analyses explained |
| 6 | Statistical methods: main analysis | Describe statistical methods and statistics used |  |  |
|  | a) | Describe how quantitative variables were handled in the analyses (i.e., scale, units, model) | NA |  |
|  | b) | Describe how genetic variants were handled in the analyses and, if applicable, how their weights were selected | 6 | All IVs were clumped for linkage disequilibrium (LD) (R^2^< 0.001; distance = 10,000 kb) to reduce the impact of correlations between SNPs |
|  | c) | Describe the MR estimator (e.g. two-stage least squares, Wald ratio) and related statistics. Detail the included covariates and, in case of two-sample MR, whether the same covariate set was used for adjustment in the two samples | 6  NA | The fixed-effect inverse variance-weighted (IVW) method and the Wald ratio method |
|  | d) | Explain how missing data were addressed | NA | We excluded plasma proteins for which instrumental variables could not be extracted, leaving 3,433 plasma proteins for further analysis. |
|  | e) | If applicable, indicate how multiple testing was addressed | 7 | To identify more actionable plasma proteins during multiple comparisons, we applied false discovery rate (FDR) correction, setting the FDR threshold at <0.2. |
| 7 | Assessment of assumptions | Describe any methods or prior knowledge used to assess the assumptions or justify their validity | 6 | Only independent SNPs (r^2^= 0.001; distance = 10,000 kb), strongly associated (*P <* 5 × 10^-8^) with the blood level of each protein, were used (Supplement Table 1 ). |
| 8 | Sensitivity analyses and additional analyses | Describe any sensitivity analyses or additional analyses performed (e.g. comparison of effect estimates from different approaches, independent replication, bias analytic techniques, validation of instruments, simulations) | 6  6-9 | The Cochran Q test was used to assess heterogeneity in causal effects, and the MR-Egger intercept was employed to evaluate horizontal pleiotropy(Supplement Table 3).  Genetic correlation analyses;  Colocalization analyses;  Functional Enrichment Analysis and pathway Analysis;  Protein-Protein Interaction Network Analysis; |
| 9 | Software and pre-registration |  |  |  |
|  | a) | Name statistical software and package(s), including version and settings used | 7 | The MR analysis analyses were performed using R software version 4.3.3 and the TwoSampleMR package. |
|  | b) | State whether the study protocol and details were pre-registered (as well as when and where) |  | NA |
|  | RESULTS |  |  |  |
| 10 | Descriptive data |  |  |  |
|  | a) | Report the numbers of individuals at each stage of included studies and reasons for exclusion. Consider use of a flow diagram |  | Exposure: Supplement Table 1  Outcome: Figure 1. |
|  | b) | Report summary statistics for phenotypic exposure(s), outcome(s), and other relevant variables (e.g. means, SDs, proportions) |  | Exposure: Supplement Table 1.  Outcomes: Supplement Table 3. |
|  | c) | If the data sources include meta-analyses of previous studies, provide the assessments of heterogeneity across these studies |  | NA |
|  | d) | For two-sample MR:  i.  Provide justification of the similarity of the genetic variant-exposure associations between the exposure and outcome samples  ii.  Provide information on the number of individuals who overlap between the exposure and outcome studies |  | We performed Cochran's Q-test to evaluate heterogeneity across the cohorts and found minimal heterogeneity for the variants and outcomes analyzed (see Supplement Table 3). Because we used summary-level statistics, we couldn't identify individuals who were included in both the exposure and outcome groups. |
| 11 | Main results |  |  |  |
|  | a) | Report the associations between genetic variant and exposure, and between genetic variant and outcome, preferably on an interpretable scale |  | Figure 2 for main analyses and Supplement Table 3,5,6,8. |
|  | b) | Report MR estimates of the relationship between exposure and outcome, and the measures of uncertainty from the MR analysis, on an interpretable scale, such as odds ratio or relative risk per SD difference |  | Figure 2, Supplement Table 3,5,6,8 for main analyses. |
|  | c) | If relevant, consider translating estimates of relative risk into absolute risk for a meaningful time period | NA |  |
|  | d) | Consider plots to visualize results (e.g. forest plot, scatterplot of associations between genetic variants and outcome versus between genetic variants and exposure) |  | Figure 2. |
| 12 | Assessment of assumptions |  |  |  |
|  | a) | Report the assessment of the validity of the assumptions | 10-11 | Methods to assess the robustness of MR findings: Cochran's Q statistical test and MR-Egger intercept |
|  | b) | Report any additional statistics (e.g., assessments of heterogeneity across genetic variants, such as I2, Q statistic or E-value) | 10-11 | In the MR analyses, we observed heterogeneity for all analyses that included two or more SNPs. We also reported Cochran's Q statistic for the results of the respective SNPs included in the analyses. |
| 13 | Sensitivity analyses and additional analyses |  |  |  |
|  | a) | Report any sensitivity analyses to assess the robustness of the main results to violations of the assumptions | 10-11 |  |
|  | b) | Report results from other sensitivity analyses or additional analyses |  |  |
|  | c) | Report any assessment of direction of causal relationship | 9 | We conducted two-sample Mendelian randomization (MR) analyses, where an odds ratio (OR) greater than 1 indicates a risk factor, and an OR less than 1 indicates a protective factor. |
|  | d) | When relevant, report and compare with estimates from non-MR analyses | 13-17 |  |
|  | e) | Consider additional plots to visualize results |  | Genetic correlation analyses are presented in Supplement Table 4 and Figure 3. Colocalization analyses are presented in Supplement Table 7 and Figure 4,S1,S2. |
|  | DISCUSSION |  |  |  |
| 14 | Key results | Summarize key results with reference to study objectives | 12 | In our MR study, after FDR correction, we identified 113 plasma proteins closely associated with sepsis and 2 plasma proteins closely associated with 28-day sepsis mortality. |
| 15 | Limitations | Discuss limitations of the study, taking into account the validity of the IV assumptions, other sources of potential bias, and imprecision. Discuss both direction and magnitude of any potential bias and any efforts to address them | 18-19 |  |
| 16 | Interpretation |  |  |  |
|  | a) | Meaning: Give a cautious overall interpretation of results in the context of their limitations and in comparison with other studies | 13-18 |  |
|  | b) | Mechanism: Discuss underlying biological mechanisms that could drive a potential causal relationship between the investigated exposure and the outcome, and whether the gene-environment equivalence assumption is reasonable. Use causal language carefully, clarifying that IV estimates may provide causal effects only under certain assumptions | 12 | Considering the crucial role of proteins in drug target development and their potential influence on the risk of sepsis and sepsis-related mortality, it is biologically reasonable to explore the causal relationship between plasma proteins and sepsis. |
|  | c) | Clinical relevance: Discuss whether the results have clinical or public policy relevance, and to what extent they inform effect sizes of possible interventions | 13-18 |  |
| 17 | Generalizability | Discuss the generalizability of the study results (a) to other populations, (b) across other exposure periods/timings, and (c) across other levels of exposure | NA |  |
|  | OTHER INFORMATION |  |  |  |
| 18 | Funding | Describe sources of funding and the role of funders in the present study and, if applicable, sources of funding for the databases and original study or studies on which the present study is based | 20 | This research was funded by the Jinhua Hospital Affiliated to Zhejiang University in 2022 (JY2022-2-05), and the Jinhua Science and Technology Research Program in 2023 (grant number 2023-4-074). |
| 19 | Data and data sharing | Provide the data used to perform all analyses or report where and how the data can be accessed and reference these sources in the article. Provide the statistical code needed to reproduce the results in the article, or report whether the code is publicly accessible and if so, where | 5-6 | Since this study utilized publicly available GWAS summary data, those interested in further details can contact the corresponding authors for additional information |
| 20 | Conflicts of Interest | All authors should declare all potential conflicts of interest | 28 | The authors declare that they have no competing interests. |
